# Antioxidant and chemoprotective potential of *Streptomyces* *levis* strain isolated from human gut

**DOI:** 10.1186/s13568-023-01570-7

**Published:** 2023-07-07

**Authors:** Jaya Verma, Shivani Attri, Saroj Arora, Rajesh Kumari Manhas

**Affiliations:** 1 Department of Microbiology, Guru Nanak Dev University, Amritsar, Punjab India; 2 Department of Botanical and Environmental Sciences, Guru Nanak Dev University, Amritsar, Punjab India

**Keywords:** Antioxidants, Cytotoxicity, Mitochondrial membrane potential, Reactive oxygen species, *Streptomyces* spp.

## Abstract

In the current study, *Streptomyces* *levis* strain HFM-2 has been isolated from healthy human gut. *Streptomyces* sp. HFM-2 was identified based on the polyphasic approach that included cultural, morphological, chemotaxonomical, phylogenetic, physiological, and biochemical characteristics. 16S rRNA gene sequence of strain HFM-2 exhibited 100% similarity with *Streptomyces* *levis* strain 15423 (T). The EtOAc extract of *Streptomyces* *levis* strain HFM-2 showed potential antioxidant activity, along with 69.53 ± 0.19%, 64.76 ± 0.13%, and 84.82 ± 0.21% of scavenging activity for ABTS, DPPH, and superoxide radicals, respectively at 600 µg/mL. The IC_50_ values i.e. 50% scavenging activity for DPPH, ABTS, and superoxide radicals were achieved at 497.19, 388.13, and 268.79 (µg/mL), respectively. The extract's reducing power and total antioxidant capacity were determined to be 856.83 ± 0.76 and 860.06 ± 0.01 µg AAE/mg of dry extract, respectively. In addition, the EtOAc extract showed protection against DNA damage from oxidative stress caused by Fenton’s reagent, and cytotoxic activity against HeLa cervical cancer, Skin (431) cancer, Ehrlich-Lettre Ascites-E (EAC) carcinoma, and L929 normal cell lines. The IC_50_ values against HeLa, 431 skin, and EAC carcinoma cell lines were found to be 50.69, 84.07, and 164.91 µg/mL, respectively. The EtOAc extract showed no toxicity  towards L929 normal cells. In addition, flow cytometric analysis exhibited reduced mitochondrial membrane potential (MMP), and enhanced levels of reactive oxygen species (ROS). The EtOAc extract was chemically analyzed using GCMS to determine the components executing its bioactivities.

**Supplementary Information:**

The online version contains supplementary material available at 10.1186/s13568-023-01570-7.

## Introduction

The generation of reactive oxygen species (ROS) is continuous during aerobic metabolism causing oxidative damage, mitochondrial disruption, dysfunction of the membrane, DNA disintegration, and ultimately ion leakage into intercellular space (Karimi and Noori 2022; Attri et al. 2022). At moderate and low concentrations, ROS plays a significant signaling molecule role in mitogenesis, or as a host defense mechanism during infections (Ser et al. 2016a, b). Oxidative damage occurs due to the irregular synthesis of ROS causing stress within the intracellular environment. This situation is considered oxidative stress, that may alter cellular function and structure. It may induce DNA denaturation or somatic mutations or resulting in protein and lipid modifications, ultimately enhancing neoplastic transformation risk (Dizdarogl 2015). In order to antagonize oxidative stress, a significant role of antioxidants exist in survival of aerobic species. They are capable of blocking or delaying the oxidant initiated oxidative damage by sharing hydrogen atoms or therefore, inhibiting the chain reactions (Apak et al. 2016). An antioxidant may pass through various pathways like prevention of free radical formation, disruption of oxidation chain reactions, singlet oxygen quenching, chelation of metal pro-oxidants, and reduction of ROS into stable compounds (Tan et al. 2015; Zhang and Tsao 2016). Antioxidants prevent negative effects on organs, tissues, and cells, associated with diabetes, cancer, and neurodegenerative and cardiovascular diseases (Kemung et al. 2020). Among these diseases, the second leading cause of worldwide death is cancer that contributes significantly to global death. The increasing population, aging, and adopted lifestyle, all contribute to an increase in cancer prevalence (Torre et al. 2016; Siegel et al. 2022). More than 10 million cancer cases per year were reported by World Health Organization in May 2018 (Zhang et al. 2020). According to the reports, cervical cancer is the main public health problem worldwide and in women it is the second most common cancer caused primarily by human-papillomavirus (HPV) infections (Scarinci et al. 2010).

Many beneficial substances that are significant in sectors like pharmacy, medicine, and biochemistry have intriguing sources in nature (Karikas 2010). Several studies have reported that natural antioxidants from plants are preferable but according to other reports microorganisms can also be used to produce natural antioxidants (Ser et al. 2015a, b; Law et al. 2017). In the microorganisms, actinobacteria have been extensively studied for microbial drug discovery because of their capacity to generate a variety of bioactive secondary metabolites (Sharma and Shah 2014). Particularly, *Streptomyces,* which is proposed by Waksman and Henrici (1943) and consists of about 780 species of Gram-positive bacteria with valid published names (Azman et al. 2015; Balasubramanian et al. 2018), has a remarkable contribution to mankind. *Streptomyces* are major producers of several chemicals with significant biological properties including antioxidant, anticancer, immunosuppressive and antimicrobial in addition to antibiotics (Rashad et al. 2015; Ser et al. 2016a, b; Lyu et al. 2017). This genus produces 75% or more naturally occurring antibiotics (Kinkel et al. 2014; Gozari et al. 2018). The primary resource of these bacteria is soil (Paderog et al. 2020). Various actinobacteria are known to form direct association with vertebrates (Tae et al. 2005). The streptomycetes presence in the healthy human gut microbiome was overlooked for a long time, inspite of their presence in various animals. The human tissue colonization of streptomycetes was underestimated due to insufficient selective techniques for the cultivation of streptomycetes, their slower growth rates, and extensive clinical microbiologist belief that they are an air-borne contamination (Herbrík et al. 2020). Recently the occurrence of microbiome (streptomycetes) in healthy skin, uterus, and respiratory tract of human has been confirmed by molecular data (Huang et al. 2015; Collado et al. 2016). However, it is found that in comparison to other animals, human guts accommodate considerably a smaller number of streptomycetes than that can be seen in other closely related animals (Bolourian and Mojtahedi 2018).

In the process of screening for different bioactivities, *Streptomyces* spp. were isolated from human stool samples through serial dilution, and evaluated for different biological activities. In this study, *Streptomyces* *levis* strain HFM-2 is being reported for potential antioxidant activity, and cytotoxicity against different cancer cell lines.

## Materials and methods

### Sample collection and isolation of potential isolate

For the isolation of actinobacteria, stool samples were collected from healthy human host after taking their informed consent in the laboratory of Dr. Sukhraj Kaur, Department of Microbiology, G.N.D.U., Amritsar. The study was approved by the Institutional Human Ethics Committee, G.N.D.U. The stool samples were collected in sterilized bags. The incubation of collected samples was done for 3 to 5 days at 28 °C. Then 100 µL of serially diluted samples (10^–3^ to 10^–6^) were spread on the surface of starch casein nitrate agar (SCNA) medium. The antibiotics, cycloheximide (50 µg/mL) and nalidixic acid (50 µg/mL) were supplemented into the medium to prevent fungal and bacterial growth, respectively. The incubation of inoculants was done at 28 °C for 7–21 days. Various colonies of actinobacteria were isolated and subcultured individually using same medium (SCNA). The isolates were stored in 20% glycerol (v/v) as spore suspension and mycelia fragments at − 20 °C.

### Characterization of *Streptomyces levis* strain HFM-2

#### Phenotypic characterization

Several cultural and morphological properties are highly distinguishing, significant, and beneficial in the genus *Streptomyces* classification*.* The characteristic properties were detected by inoculation of isolate HFM-2 on SCNA and various International *Streptomyces* Project (ISP) media (Shirling and Gottlieb 1966). The color of cultural colony i.e. aerial and substrate mycelia, sporulation and diffusible pigment production were monitored, and described using standard ISCC-NBS color charts (Pridham 1965). The morphology was examined under a light microscope. The isomeric forms of diaminopimelic acid, sugars in cell wall and whole-cell hydrolysate were examined according to Becker et al. (1964).The utilization of sugars as a carbon source was investigated (Shirling and Gottlieb 1966). Physiological tests were carried out by growing the culture on SCNA at various pH (2.0–12.0), temperatures (15–45 °C), and NaCl concentrations (0–10% w/v). Various biochemical tests (hydrolysis of casein, gelatin and starch, methyl-red and Voges-Proskauer tests, indole production, and oxidase production were performed according to Barrow and Feltham (1965). Nitrate reduction, catalase production, citrate utilization, and urea hydrolysis were determined according to Lányi (1988) and Barrow and Feltham (1965).

#### Molecular and phylogenetic analysis

For the molecular identification (16S rRNA gene sequencing), the extraction of genomic DNA was done according to Marmur (1961), and amplification process was performed by polymerase chain reaction (PCR) using primers 1492r (5’-AG AAAGGAGGTGATCCAGGC-3′) and 27f (5’-AG AGTTTGATCCTGGCTCAG-3′). QIA quick gel extraction kit (Qiagen, Germany) was used to purify PCR amplified product. The PCR purified product was sequenced from the Institute of Microbial Technology (IMTECH), Chandigarh, India. The obtained sequence (1375 bp) was aligned with the other closely related taxonomic group sequences from EzTaxon database using Clustal W Program (Chun et al. 2007). The neighbour-joining method was used to construct phylogenetic tree with bootstrap values based on 1000 replications using the MEGA X.0 software (Tamura et al. 2013).

### Extraction of metabolites

For the production of antioxidant metabolites, the production medium was inoculated with *Streptomyces* culture, and fermentation process was carried out in Erlenmeyer flasks on a rotary shaker. The actinobacterial culture of 7 days was inoculated in SCN broth (100 mL) to prepare the seed culture. After 48 h, the aseptically transfer of inoculum 2% was done in 250 mL Erlenmeyer flasks, each containing same seed medium (50 mL) and incubated for five days at 28 °C with 180 rpm shaking. Following fermentation, the centrifugation of broth was done at 10,000×*g* for 20 min at 4 °C to separate the supernatant. For bioactive metabolites extraction, the supernatant was extracted thrice with 1:1 (supernatant:solvent) ratio of ethyl acetate. Further the organic phase was concentrated to dryness at 50 °C using rotavapor (BUCHI). Prior to bioactivity screening assays, the EtOAc extract was redissolved in ethyl acetate.

### Antioxidant activity

#### DPPH free radical scavenging assay

The antioxidant activity of the EtOAc extract was determined using DPPH (2, 2-diphenyl-1-picrylhydrazyl) assay, which was modified slightly from the method described by Kumar et al. (2014). Freshly prepared 0.002% (w/v) DPPH (Hi-Media) in methanol was mixed with extract. The reaction mixture was incubated in dark at room temperature for 30 min, Further microplate reader was used to measure absorbance at 517 nm. As a positive control, vitamin C was used. The % inhibition of DPPH free radicals was calculated using the formula:

$${\text{DPPH}}\;{\text{Scavenging}}\;{\text{Activity}}\;\left( \% \right) = \left[ {\left( {{\text{A}}_{{\text{o}}} {-}{\text{ A}}_{{\text{e}}} } \right)/{\text{A}}_{{\text{o}}} } \right] \times {1}00$$

where, A_o_ is the absorbance of the control and A_e_ is the absorbance of the test extract/ standard.

#### ABTS free radical scavenging assay

The free radical scavenging assay with 2, 2′-azino-bis (3-ethylbenzothiazoline-6-sulfonic acid) (ABTS) was carried out using the method reported by Re et al. (1999) with small modification. ABTS free radical solution (190 µL) was tested against a series of varying concentrations of extract in a 96-well microtiter plate. The reaction of ABTS stock solution (7 mM) and potassium persulphate (2.45 mM) for 24 h in dark result in formation of ABTS free radical solution. Before performing the experiment, the dilution of ABTS radical solution was done upto absorbance of 0.7 ± 02. Further, at room temperature the reaction mixture was left in dark for 3 min before measuring the absorbance at 743 nm. As a positive control, ascorbic acid was employed. The ABTS radical inhibition activity was expressed as a percentage.

$${\text{ABTS}}\;{\text{Scavenging}}\;{\text{Activity}}\;\left( \% \right) = \left[ {\left( {{\text{A}}_{{\text{o}}} {-}{\text{ A}}_{{\text{e}}} } \right)/{\text{A}}_{{\text{o}}} } \right] \times {1}00$$

where A_o_ is the absorbance of the control and A_e_ is the absorbance of the test extract/standard.

#### Superoxide anion scavenging assay

The superoxide radical scavenging assay was performed spectrophotometrically by measuring the reduction of NBT using the method by Nishikimi et al. (1972) with some modifications. All the mixture solutions were prepared in phosphate buffer (pH 7.4). The final solution was made by combining 1 mL of NBT (156 M), 1 mL of NADH (468 M), and 1 mL of extract (100–600 µg/mL). The reaction was started by adding 1 mL of PMS (60 M), and the mixture was incubated at 25 °C for 5 min before measured at 560 nm. Ascorbic acid was utilised as a reference, and % inhibition was determined using the formula:

$${\text{Superoxide}}\;{\text{Anion}}\;{\text{Scavenging}}\;{\text{Activity}}\;\left( \% \right) = \left[ {\left( {{\text{A}}_{{\text{o}}} {-}{\text{A}}_{{\text{e}}} } \right)/{\text{A}}_{{\text{o}}} } \right] \times {1}00$$

where A_o_ is the absorbance of the control and A_e_ is the absorbance of the test extract/standard.

#### Ferric reducing antioxidant potential (FRAP) assay

The total ferric reducing activity was carried out by FRAP assay according to the method described by Oktay et al. (2003). The blue color complex formation with trichloroacetic acid (C_2_HCl_3_O_2_), potassium ferricyanide (K_3_Fe(CN)_6_), and ferricchloride (FeCl_3_) was measured. Potassium ferricyanide (2.5 mL, 1% w/v) and Phosphate buffer (2.5 mL, 0.2 M, pH 6.6) were added and reacted to 1 ml of extract (1 mg/mL). After addition of trichloroacetic acid (2.5 mL, 10% w/v), the mixture was incubated at 50 °C for 20 min. For 10 min, the resultant mixture was centrifuged at 10000×*g*. Before adding FeCl_3_ (500 µL, 0.1% w/v), the recovered supernatant (2.5 mL) was combined with distilled water (2.5 mL). At 700 nm, the absorbance was measured and compared to vitamin C.

#### Phosphomolybdenum assay

The total antioxidant capacity (TAC) or molybdate ion reduced by extract was evaluated according to the method proposed by Prieto et al. (1999) with small changes. 100 µL of extract was mixed with 900 μL of reagent solution (28 mM sodium phosphate, 4 mM ammonium molybdate, and 0.6 M sulphuric acid). The mixture incubation was done for 90 min at 95 °C in a water bath, then cooled at room temperature. The absorbance was measured at 695 nm. From the standard curve, the total antioxidant capability was determined and reported as mg/Ascorbic Acid Equivalents (AAE)/mg dry weight of extract.

### Estimation of total phenolic content (TPC)

The TPC content in the EtOAc extract was estimated using Folin–Ciocalteau method in 96-well microtitre plate according to Yu et al. (2002) with slight modifications. 100 µL of Folin–Ciocalteau reagent were combined with 10 µL of the EtOAc extract (1 mg/mL) and incubated for 5 min at room temperature. After incubation, 80 µL of 7.5% sodium carbonate (Na_2_CO_3_) was added in each well followed by 30 min incubation at room temperature in the dark. At 750 nm, the absorbance was measured using a microplate reader. The amount of TPC was calculated as mg of Gallic Acid Equivalents (GAE)/mg of the extract using the calibration curve of gallic acid.

### Estimation of total flavonoid content (TFC)

The TFC content of EtOAc extract was quantified as described by Tan et al. (2017) using the 96 well plate method. 100 μL of distilled water was added into each of the well, followed 10 μL of 5% sodium nitrite (NaNO_2_) and 25 μL of the EtOAc extract (1 mg/mL). After 5 min of incubation at room temperature, 15 μL of 10% aluminium chloride (AlCl_3_) was added and the reaction mixture was allowed to stand for 6 min before the addition of 50 μL of 1 M NaOH. Following, 50 μL of double distilled water was added to each well and mixed properly. The absorbance at 510 nm was taken immediately using microplate reader. Catechin was used as positive control and used for plotting calibration curve. The total flavonoid content (TFC) was calculated as the equivalent of catechin per/mg of dry weight of the extract.

### Determination of phenolic compounds using UHPLC

To analyse the phenolic compounds in the extract, ultra-high performance liquid chromatography (UHPLC) was carried out using Nexera UHPLC (Shimadzu) systems. The apparatus included an LC30 AD quaternary gradient pump, an SPD-M20 A diode array detector (DAD), a CBM-20 A communication bus module, a CTO-10 AS VP column oven, a DGU-10 A5 prominence degasser, C-18 column of dimensions 150 × 4.6 × 5 μM particle size, SIL-30 AC Nexera auto sampler, and 70% methanol used as mobile phase with combination of milli Q water at flow rate of 1 mL/min. The run time of the sample was 25 min and, column temperature was maintained at 30 °C. All constituents were identified by comparison with standards.

### DNA protective activity

This assay was carried out in accordance with the instructions provided by Lee et al. (2007). This approach was used to assess the ability of *Streptomyces* *levis* strain HFM-2 extract to protect supercoiled pBR322 plasmid against the hydroxyl radicals of Fenton’s reagent. The reaction mixture included 2 µL of plasmid DNA (50 µg/100 µL), 10 µL of Fenton's reagent (80 µM FeCl_3,_ 30 mM H_2_O_2_, and 50 µM ascorbic acid), various concentrations of EtOAc extract (2–10 µg/mL) and MQ water upto 20 µL. In negative control, an equivalent volume of MQ water was added in place of Fenton's reagent. Rutin was employed as a positive control. It was incubated for 30 min at 37 °C. DNA analysis was done using 1% agarose gel electrophoresis.Quantitative analysis of DNA damage was done by using Image j software. The percentage of native supercoiled (Form I), single-stranded nicked (Form II), and linear (Form III) DNA was calculated.

### Cell line maintenance and growth conditions

The HeLa cervical cancer, A43 skin cancer, (EAC) carcinoma, and L929 normal cell lines were obtained from the National Centre for Cell Science (NCCS), Pune (India), and were maintained in culture medium DMEM (Dulbecco’s modified eagle’s medium) with antibiotics (100 µg/mL streptomycin and 100 Units/mL penicillin) and 10% Fetal Bovine Serum. The cell lines were kept at 37 °C in an atmosphere with 90% relative humidity and 5% CO_2_. A bright field inverted microscope was used to observe the cultures for confluency, and the lack of bacterial and fungal contaminations (Hsu et al. 2006).

### Cytotoxic activity using MTT assay

The cytotoxicity of EtOAc extract on human cancer cell lines was assessed using the 3-(4,5-dimethylthiazol-2yl)-2,5-diphenyltetrazolium bromide (MTT) test according to the protocol described by Mosmann (1983). In the 96 well microtiter plate, cells (5000 cells/well) were seeded and allowed to adhere overnight. After that, different concentrations of EtOAc extract (31.25, 62.5, 125, 250, 500 µg/mL) were added to each well and incubated for 24 h. Following that, 100 µL of 0.5 mg/mL MTT dye (Sigma-Aldrich) was added to each well, and the plates were incubated for 4 h at 37 °C in a humidified environment with 5% CO_2_. The blue formazan crystals formed by the MTT reaction were dissolved in 100 µL of DMSO. A microplate reader was used to measure the color at 595 nm. The proliferation of cells subjected to treatment was calculated using the following formula

$${\text{Cell}}\;{\text{Viability}}\;\left( \% \right) = \left[ {\left( {{\text{A}}_{{\text{o}}} {-}{\text{ A}}_{{\text{e}}} } \right)/{\text{A}}_{{\text{o}}} } \right] \times {1}00$$

where A_o_ denotes the absorbance of untreated cells (only medium) and A_e_ denotes the absorbance of treated cells (with extract).

### Mechanistic study

#### Brightfield microscope

HeLa cells (2 × 10^5^/well) were cultured in a 24-well plate for 24 h to examine the morphological changes in the cells. Following that, cells were treated with different concentrations of EtOAc extract (31.25, 62.5, 125, 250, 500 µg/mL) and after 24 h morphological alterations in cells were observed.

#### Acridine orange/ethidium bromide-based staining

The morphological changes in the HeLa cells were examined using the AO/Etbr double staining method as described by Leite et al. (1999). Cells were seeded into each well and incubated for 24 h in 5% CO_2_ humidity, at 37 °C. The seeded cells were treated with different concentrations of EtOAc extract and stained with mixture of 1 μL AO (100 µg/mL, A6014, Sigma, Germany) and 1 μL EB (100 µg/mL, E7637, Sigma, Germany) diluted with PBS (pH 7.4) for 5 min. Then 24 µL of cell suspension (0.5 × 10^6^ cells/mL) was placed on clean glass slide and was immediately examined under a fluorescence microscope.

#### DAPI staining

The 4’,6-diamidino-2-phenylindole (DAPI) dye was used to evaluate the nuclear morphology of HeLa cells according to Morikawa and Yanagida (1981). The HeLa cells were exposed to EtOAc extract after growing to a density 4 × 10^5^ cells per well in a six-well plate for 24 h. The cells were fixed with 4% cold paraformaldehyde (PFA) in the dark for 15 to 20 min at room temperature and washed with PBS. After that, cells were stained with 4, 6-diamidino-2-phenylindole DAPI (10 µg/mL) and left for 30 min at room temperature. The nuclear morphology was acquired using a Nikon eclipse TiE inverted fluorescent microscope at a magnification of 100 X.

### Reactive oxygen species (ROS) analysis by flow cytometer

Estimation of ROS content in HeLa cells was carried out with 2', 7'- Dichlorodihydrofluoresceindiacetate (DCFH-DA), chemiluminescent dye as described by Kuete et al. (2014). HeLa cells (4 × 10^5^ cells/well) were poured in a six-well plate and treated with EtOAc extract at different concentrations (22.43, 99.78, and 443.86 µg/mL) for 24 h. Then, cells were allowed to react with the dye (10 μM) for 30 min at 37 °C, followed by harvesting and washing with 1 × PBS. The oxidative stress was observed by flow cytometer (BD AccuriTMC).

### Mitochondrial membrane potential analysis

The alteration in MMP in HeLa cells was determined according to the method described by Wang et al. (2012) with some modification. HeLa cells were seeded at a density of 1.2 × 10^4^ to 1.5 × 10^4^ cells/well in a 96 well plate and treated with different concentrations (22.43, 99.78, and 443.86 µg/mL) of EtOAc extract for 24 h to investigate MMP loss. After treatment, cells were washed, incubated with 5 μg/mL of rhodamine 123 for 30 min at 37 °C and washed with PBS. Then, cells were centrifuged, and pellet was further suspended in 1 × PBS. After that, the fluorescence of cells was analyzed using a flow cytometer (BD AccuriTMC 6).

### GCMS analysis

The EtOAc extract was subjected to GC–MS analysis on THERMO Trace 1300GC coupled with THERMO TSQ8000 Triple Quadrupole MS (column BP-5MS).The experiment was initiated by maintaining the column temperature at 3 °C for 10 min, followed by an increase of 50 °C per minutes to 260 °C and was kept at same temperature for 5 min, carrier gas-helium, flow-rate-1.0 mL/minute, ionized voltage of 70 eV, 15:1 split ratio and mass spectrum scans range of 35–650 (m/z) were used. Comparison of the mass spectrum between detected chemical constituents and the standards available in GC–MS NIST Ver.21 library was done to identify the chemical constituents present in extract (Kaur et al. 2020).

### Statistical analysis

All the statistical analyses were performed in triplicates. Results were expressed in mean ± standard error (SE). Statistical significance was considered at p ≤ 0.05. The one-way analysis of variance (ANOVA) was used for the analysis.

## Results

### Isolation, identification, and characterization of *Streptomyceslevis* strain HFM-2

**Fig. 1:**
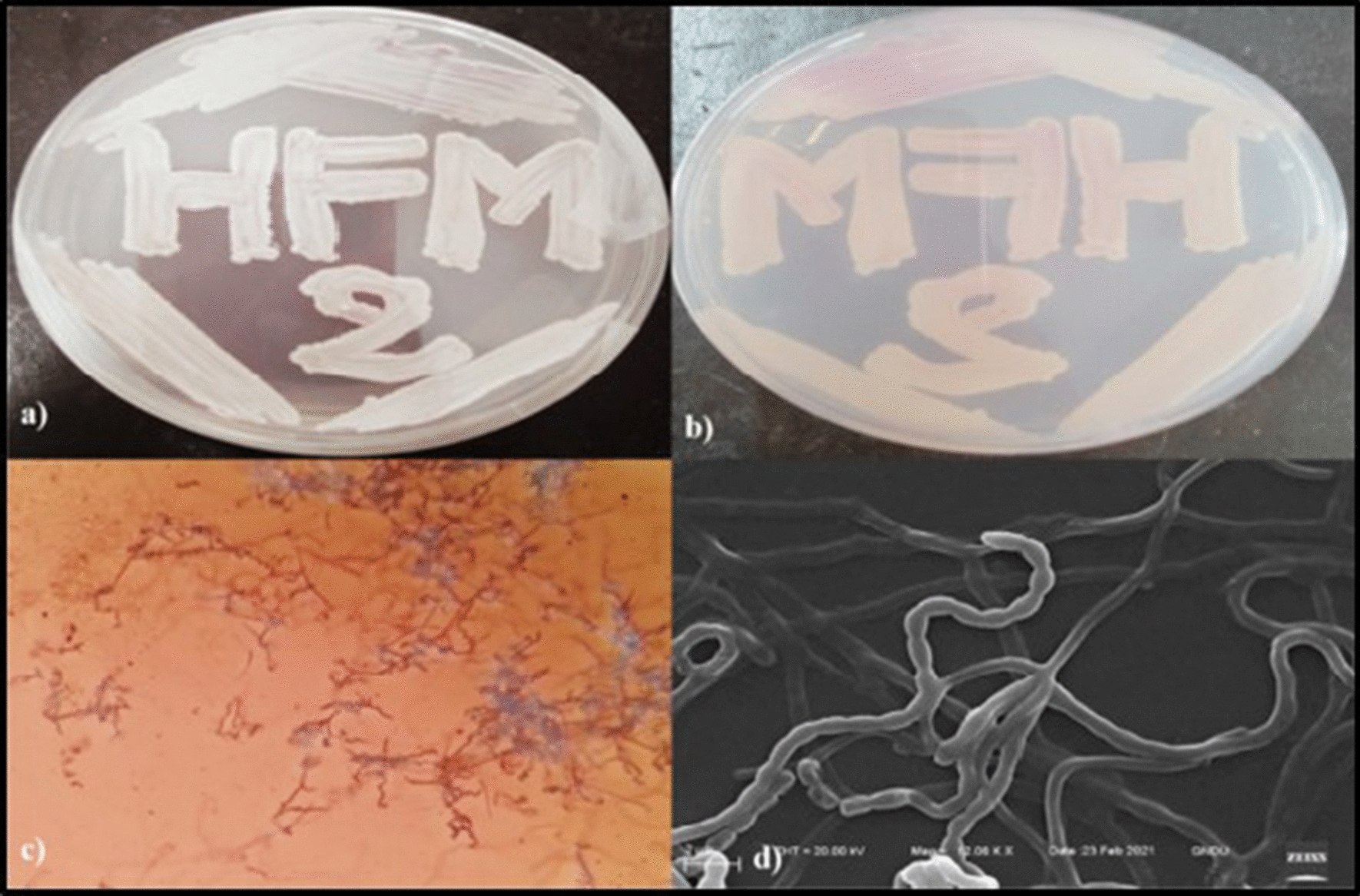
Cultural and morphological characteristics of isolate HFM-2. **a** Aerial mycelium, **b** Substrate mycelium, **c** light microscopic image at 100X bearing spiral spore chains on aerial hyphae **d** SEM image at 12000X, showing smooth surface spores

Total thirty stool samples were processed, and out of these ten actinobacterial cultures were isolated. Among these ten isolates, *Streptomyces* *levis* strain HFM-2 showed potent bioactivities including antioxidant, anticancer and antimicrobial activities; hence selected for detailed morphological, taxonomical, physiological, and biochemical studies. The strain HFM-2 produced white sporulation, light pink color aerial mycelium and dark pink color substrate mycelium on SCNA medium. Spira type spore chains (Fig. 1) were observed under light microscope (100X). SEM analysis exhibited spore chains bearing 20–23 smooth surface, cylindrical spores per chain. Well flourished cultural growth was observed on all the ISP media except ISP 2 medium (Additional file 1: Table S1 and Fig. S1). Whole-cell hydrolysates and chemotaxonomic analysis of cell wall demonstrated the presence of type 1 cell wall with LL-DAP as diagnostic amino acid without characteristic sugars. The biochemical and physiological characteristics of the isolate have been represented in Table 1. The growth was observed between 15 and 45 ºC with optimum temperature at 28 °C and pH range from 2.0 to 11.0 with optimum pH at 6.0. The NaCl tolerance was observed up to 2% concentration. The strain HFM-2 exhibited positive results for hydrolysis of starch, gelatin, citrate and urea, whereas negative results were observed for cellulase, esculin, indole production, and MR-VP test. Catalase production and oxidase test exhibited positive and negative results, respectively. Isolate HFM-2 neither reduced nitrate nor produced H_2_S. The isolate represented significant growth by utilizing carbon source in form of arabinose, xylose and glucose, weak growth was observed on rhamnose and raffinose containing medium whereas no growth was observed on lactose and trehalose containing medium (Table 1).

**Table 1:** Morphological, chemotaxonomical, physiological and biochemical characteristics of *Streptomyces* *levis* strain HFM-2:

Properties	Result
*Morphological* *characteristics*	
Spore surface	Smooth
Aerial mycelium color	Light pink
Substrate mycelium color	Dark Pink
Spore morphology	Spiral type
*Chemotaxonomical* *characteristics*	
L-L Diaminopimelic acid	+
Whole-cell sugar	–
*Physiological* *characteristics*	
Optimum pH for growth	6.0
Optimum temperature for growth	28 °C
Temperature range for growth	15 to 45 °C
pH range for growth	2.0 to 11.0
Production of melanoid pigment	(Black)
NaCl tolerance	Up to 2%
*Biochemical* *characteristics*	
MRVP	–
Starch hydrolysis	+
Cellulase production	–
Urease production	+
Esculin hydrolysis	–
Indole production	–
Citrate utilization	+
Nitrate reduction	–
Gelatin hydrolysis	–
Catalase test	+
H_2_S production	–
Oxidase test	–
*Utilization* *of* *carbon* *source*	
Arabinose	+
Xylose	+
Rhamnose	+
Glucose	+
Lactose	–
Raffinose	+
Trehalose	–

Positive = ( +), negative = (−)

### 16S rRNA gene amplification and phylogenetic analysis

For the molecular identification of selected isolate HFM-2, almost complete determination was done based on 16S rRNA gene sequence (1375 bp, deposited in GenBank under accession no. MW566167). The sequence of the strain HFM-2 displayed 98.75–100% sequence similarity with different *Streptomyces* spp. and showed the maximum similarity (100%) with *Streptomyces* *levis* NBRC 15423 (T). In phylogenetic tree, constructed using neighbor-joining method, it formed a well-defined clade among *Streptomyces* spp. and a sister species to *Streptomyces* *levis* strain NBRC 15423 (T) with a bootstrap value of 63% (Additional file 1: Fig. S2). Therefore, on the basis of phylogenetic analysis, isolate HFM-2 was designated as *Streptomyces* *levis* strain HFM-2. The culture has been deposited in an International Depository Authority, MTCC and GenBank, CSIR-IMTECH, Chandigarh (India), with the accession number MTCC-13134.

### Fermentation and extraction

The most effective solvent for achieving the highest recovery of active metabolites from the fermentation broth at pH 3.0 was revealed to be ethyl acetate. A rotary evaporator was used to further concentrate the extracted metabolites, and an orange-colored dry extract was collected and further dissolved in ethyl acetate.

### Antioxidant activity of *Streptomyces levis* strain HFM-2

**Fig. 2:**
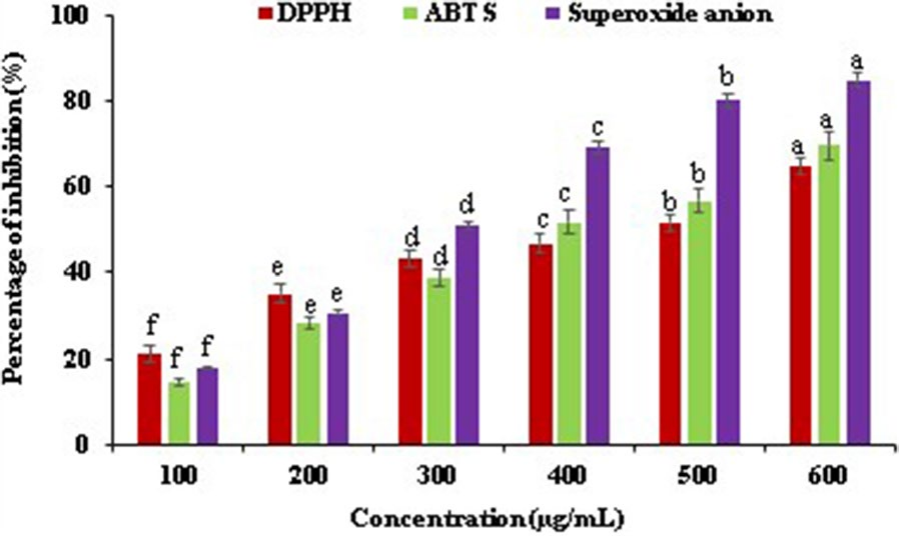
The antioxidant activities demonstrated by EtOAc extract of HFM-2 in different antioxidant assays. Data are mean ± SE of three independent experiments with each experiment conducted in triplicate at the level of significance p ≤ 0.05. Data labels with different letters represent significant differences among them

The antioxidant potential of EtOAc extract of *Streptomyces* *levis* strain HFM-2 was evaluated depending on *in-vitro* antioxidant assays such as the DPPH, ABTS, and superoxide anion radical assays. Additional file 1: Table S2 and Fig. 2 represent results based on percentage inhibition of free radicals. In this study, EtOAc extract of HFM-2 showed potential dose-dependent DPPH, ABTS and superoxide anion scavenging activities measuring (p < 0.05) with 64.76 ± 0.13%, 69.53 ± 0.19%, and 84.82 ± 0.21%, respectively at 600 µg/mL. The IC_50_ values i.e., 50% scavanging activity against DPPH, ABTS and superoxide anion were achieved at concentrations of 497.19, 388.13 and 268.79 µg/mL, respectively.

FRAP and TAC were expressed as AAE/mg dry weight of extract. The high absorbance at specific wavelength exhibits higher antioxidant capacity. The reduction potential of ferric ion and molybdate ion were measured as 856.83 ± 0.76 and 860.06 ± 0.01 µg as AAE/mg dry weight of EtOAc extract, respectively.

### TPC and TFC contents

In TPC of extract, the phenolic compounds undergo reaction in Folin–Ciocalteau reagent resulting in blue-colored complex formation in alkaline medium exhibiting good antioxidant activity. The TPC of EtOAc extract was expressed as Gallic Acid Equivalents/mg of the dry weight of extract, and measured as 77.63 ± 0.02 µg GA/mg of dry extract. The total flavonoid content of EtOAc extract was measured as 71.25 ± 0.01 µg Catechin/mg of dry extract.

### Determinination of phenolic compounds using UHPLC

**Fig. 3:**
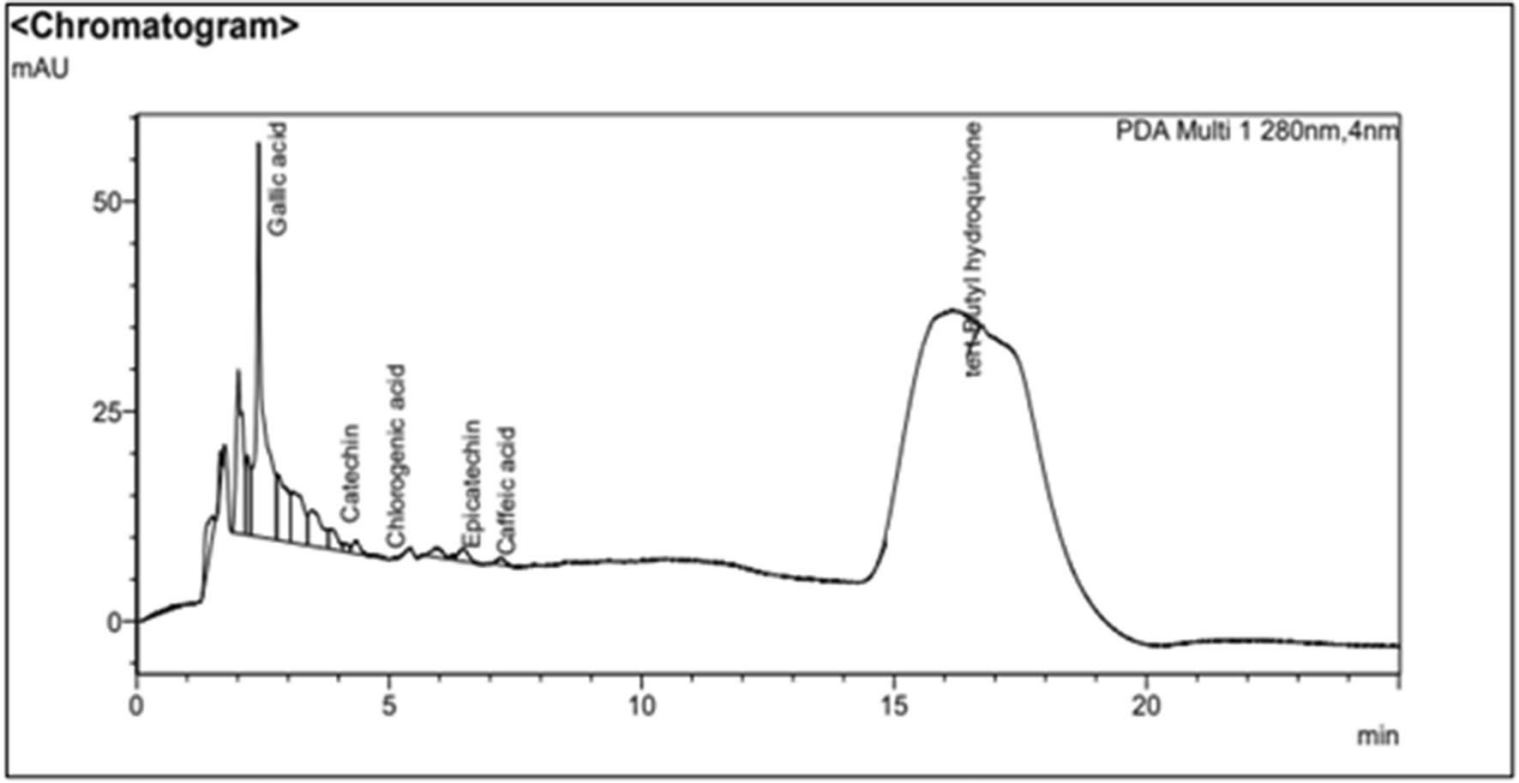
UHPLC chromatogram of *Streptomyces* *levis* strain HFM-2 EtOAc extract showing six different phenolic compounds based on particular retention time

The phenolic compounds in the EtOAc extract of *Streptomyces* *levis* strain HFM-2 were assessed by performing UHPLC analysis. Different retention times were used to identify phenolic compounds under the curve. The results showed that EtOAc extract contained six compounds i.e. gallic acid, catechin, epicatechin, caffeic acid, chlorogenic acid tert-butyl hydroquinone (Table 2 and Fig. 3**).**

**Table 2:** UPLC analysis of EtOAc extract from *Streptomyces* *levis* strain HFM-2

Sr. No.	Phenolic compounds	Retention time (min)	Molecular formula	Molecular weight (MW)
1	Gallic acid	2.416	C_7_H_6_O_5_	170.12
2	Catechin	3.867	C_15_H_14_O_6_	290.27
3	Epicatechin	6.264	C_15_H_14_O_6_	290.27
4	Caffeic acid	6.952	C_9_H_8_O_4_	180.16
5	Chlorogenic acid	4.768	C_16_H_18_O_9_	354.41
6	Tert-Butyl hydroquinone	16.176	C_10_H_14_O_2_	166.22

### DNA protective activity

**Fig. 4:**
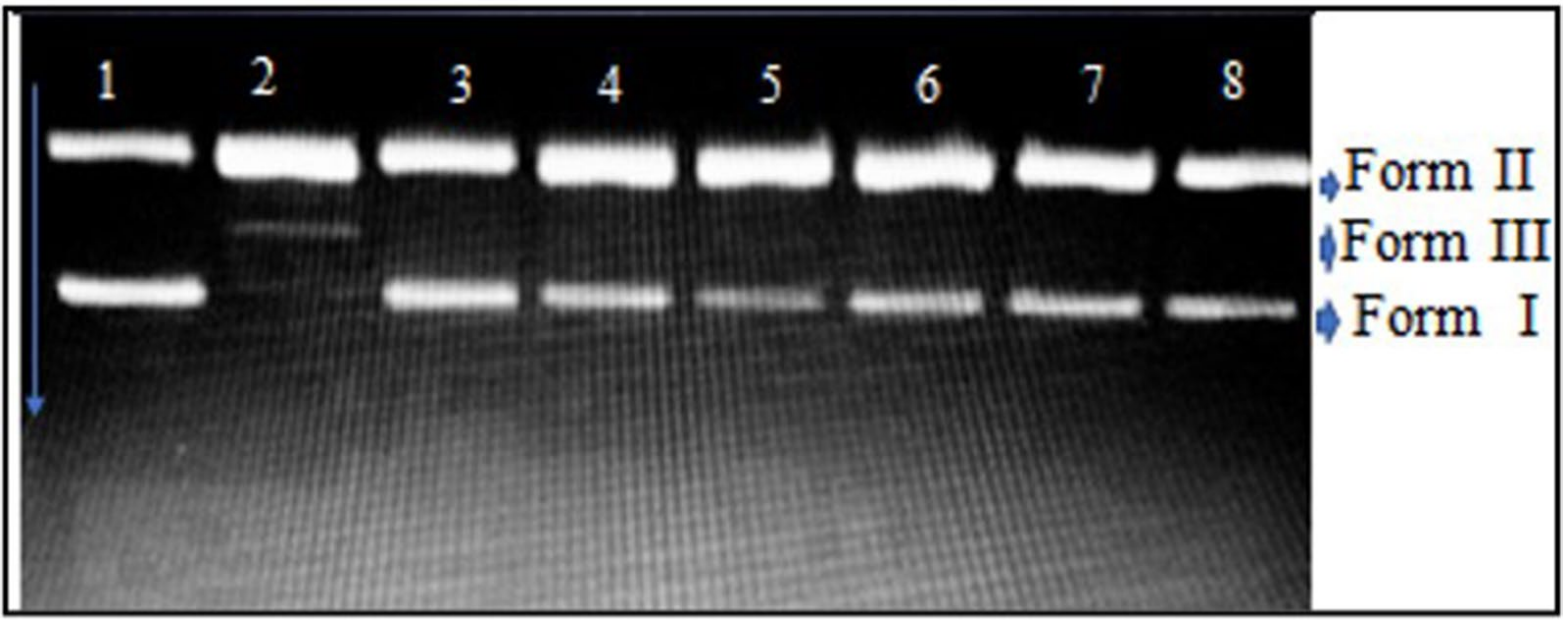
DNA Nicking assay; Lane 1: Negative control (only DNA); Lane 2: Fenton’s reagent; Lane 3: Positive control (rutin, 10 μg); and Lane 4–8: Fenton’s reagent + 2, 4, 6, 8 and 10 μg of the EtOAc extract of HFM-2; Form I = Supercoiled; Form II = Single strand nicked DNA and Form III = Linear

The DNA nicking assay revealed protective effects of EtOAc. According to the results supercoiled form of pBR322 plasmid DNA was degraded to single-stranded nicked and linear forms in the present of Fenton’s reagent. However, the addition of EtOAc extract to reaction mixture minimized the DNA damage as shown in lanes 4–8 (Fig. 4). In the presence of Fenton’s reagent and EtOAc extract (at 2–10 μg/mL) the amount of plasmid DNA was observed as 21.14%, 29.46%, 30.49% 31.49% and 37.75%, respectively via densitometric analysis (Additional file 1: Fig. S3). The results were compared with native supercoiled DNA amount present in positive control (Fenton’s reagent + rutin) with 43.11% Form I and 56.88% % Form II. However in the presence of Fenton’s reagent, Form I was found to be degraded completely with simultaneous increase of Form II from 57.39 to 78.07%, and Form III was observed as 22.90%.

### Cytotoxic activity using MTT assay

**Fig. 5:**
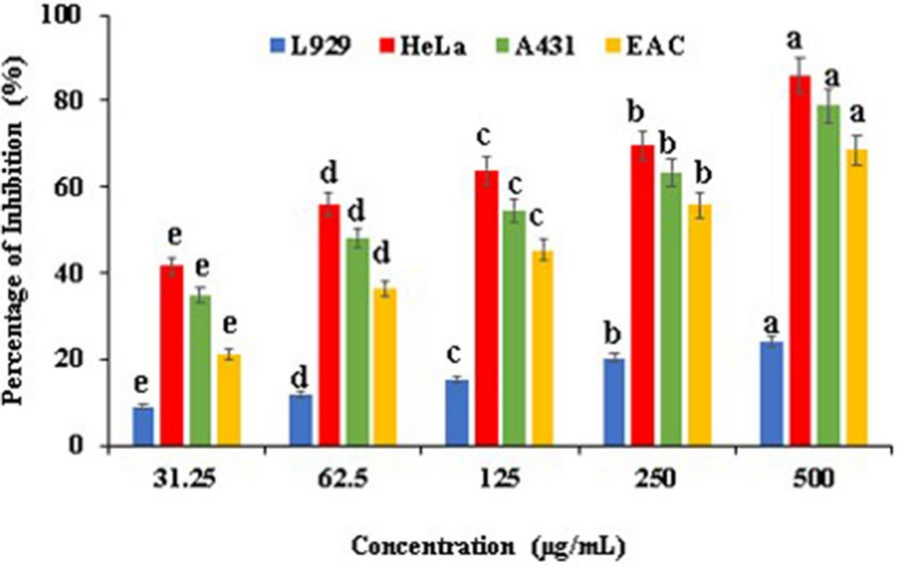
Cytotoxic activity of EtOAc extract from *Streptomyces* *levis* strain HFM-2 against different cancer cell lines using MTT assay. Data represented as mean ± SE at the level of significance p ≤ 0.05. Data labels with different letters represent significant differences among them

The EtOAc extract showed dose-dependent significant cytotoxicity against the HeLa, A431 skin, EAC carcinoma and L929 normal cell lines (Fig. 5). The EtOAc extract showed 85.93 ± 0.50%, 78 ± 81%, 68.59 ± 0.03% inhibition at concentration of 500 µg/mL against HeLa, A432 skin, EAC cells, respectively. The IC_50_ values were found to be at 50.69, 84.07, and 164.91 µg/mL, respectively. An increase in the concentration of extract increased the cytotoxicity. As for the normal cell line (L929), the EtOAc extract showed insignificant cytotoxic effect with the maximum of 24.02 ± 0.01% inhibition at the maximum concentration tested i.e. at 500 µg/mL.

### Assessment of cell morphology

**Fig. 6:**
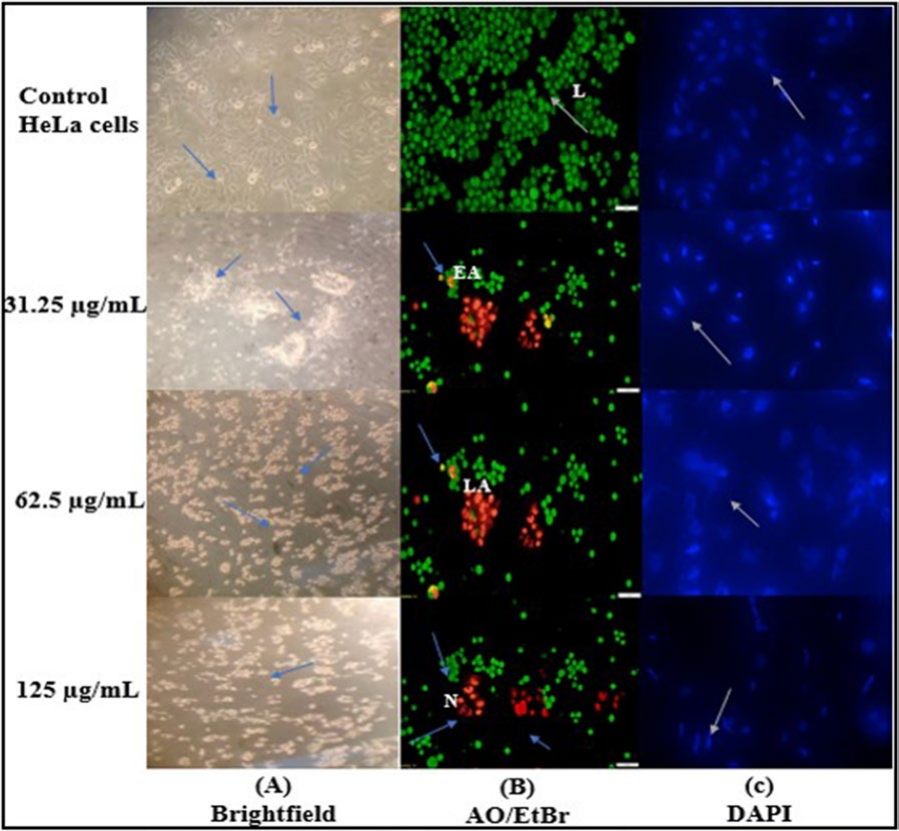
**A** Inverted brightfield images of HeLa cancer cell line, **B** Fluorescence microscopic images of acridine orange and ethidium bromide (AO/EtBr) staining; Arrow indicates live (L) cells, early apoptosis (EA), late apoptosis (LA) and necrotic (N); **C** HeLa cells stained with DAPI staining

EtOAc extract induced morphological changes in HeLa cancer cells i.e. angular to round forms which were potentially increased in a dose-dependent manner. The cells without any treatment showed angular shape (Fig. 6A).

AO/ EtBr were used to differentiate cells that are in stages of condensed nuclei, membrane blebbing, apoptosis and necrosis. They were stained green (live cells) whereas non-viable cells were stained orange (early apoptosis, late apoptosis) and red (necrotic) i.e. dead cells (Fig. 6B).

DAPI staining of EtOAc treated HeLa cells clearly showed significant changes in nuclear morphology, simultaneously apoptotic nuclei and cell death. However, no alteration in nuclear morphology was observed in bluish intact nuclei of control (Fig. 6C). Therefore, the results clearly showed that the EtOAc extract induced modification in nuclear morphology, leading to apoptotic cell death in HeLa cells.

### ROS analysis

**Fig. 7:**
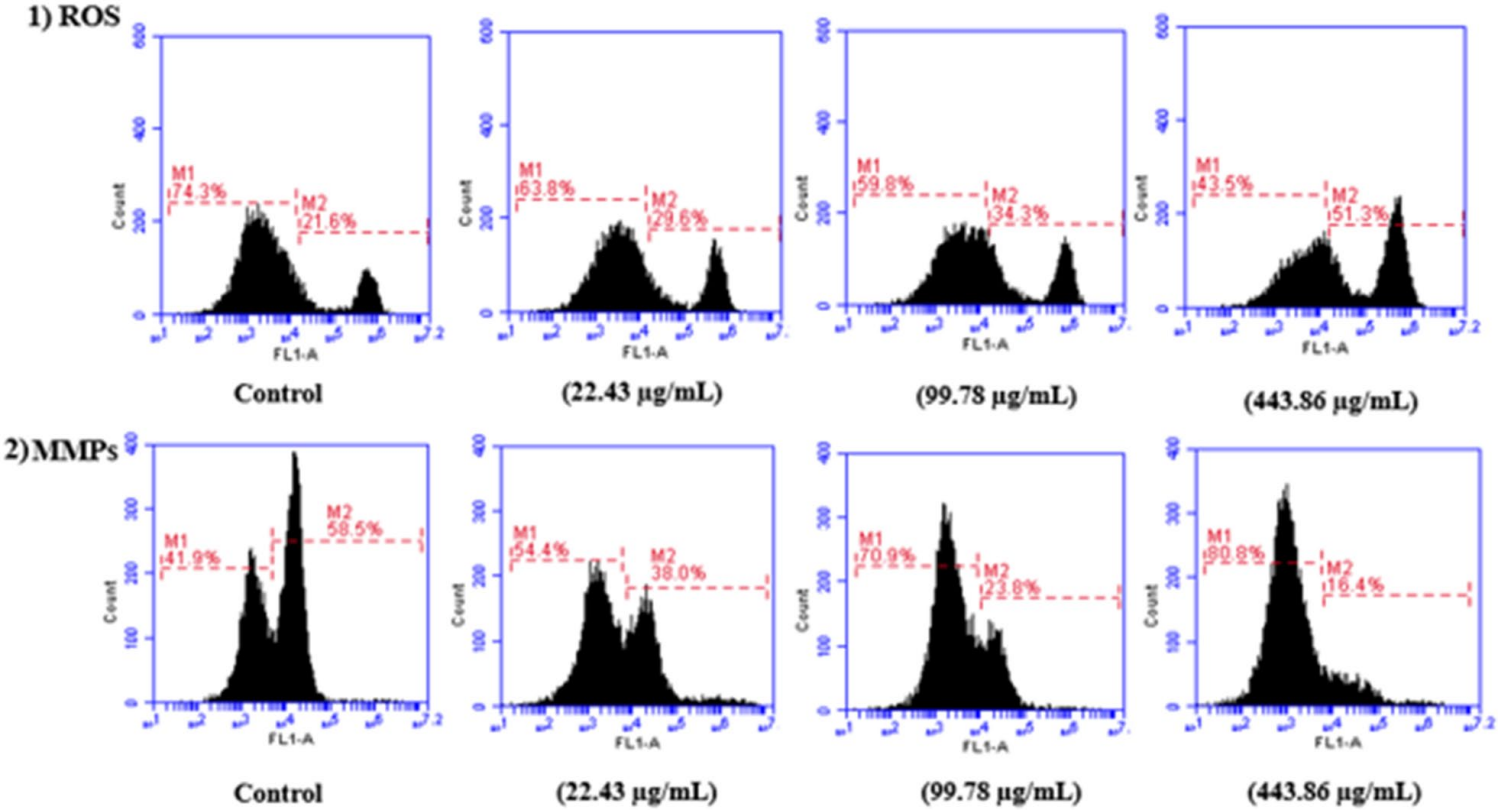
**1** The accumulation of intracellular ROS in HeLa cancer cells were detected using DCFH-DA staining (M1 represents intact cell population and M2 represents cell with the accumulation of intracellular ROS); **2** The disruption of mitochondrial membrane potential (ΔΨm) in HeLa cancer cells treated with EtOAc extract using Rhodamine 123 staining (M1 represents cells with disruption of MMP and M2 represents the intact cells)

The increment of ROS generation usually causes mitochondrial dysfunctions. Therefore, the influence of EtOAc extract on ROS production in HeLa cells was evaluated. The results demonstrated that intracellular ROS production was increased to 29.6%, 34.3%, and 51.3% at 22.43, 99.78 and 443.86 µg/mL of EtOAc extract, respectively as compared to control cells (21.6%). These results expressed that EtOAc extract induced the increment of ROS generation that might cause variations in redox state of cells causing apoptosis in HeLa cancer cells which is shown in Fig. 7(1).

### MMP loss

In order to track the variations in MMP, the effect of EtOAc extract was observed on loss of MMP in HeLa cells. The results demonstrated that EtOAc extract induced MMP loss with increasing concentration i.e. 54.4%, 70.9%, 80.8% at 22.43, 99.78, and 443.86 µg/mL which was shown in Fig. 7(2), respectively in comparison to control (41.9%) cells.

### GC–MS analysis

**Fig. 8:**
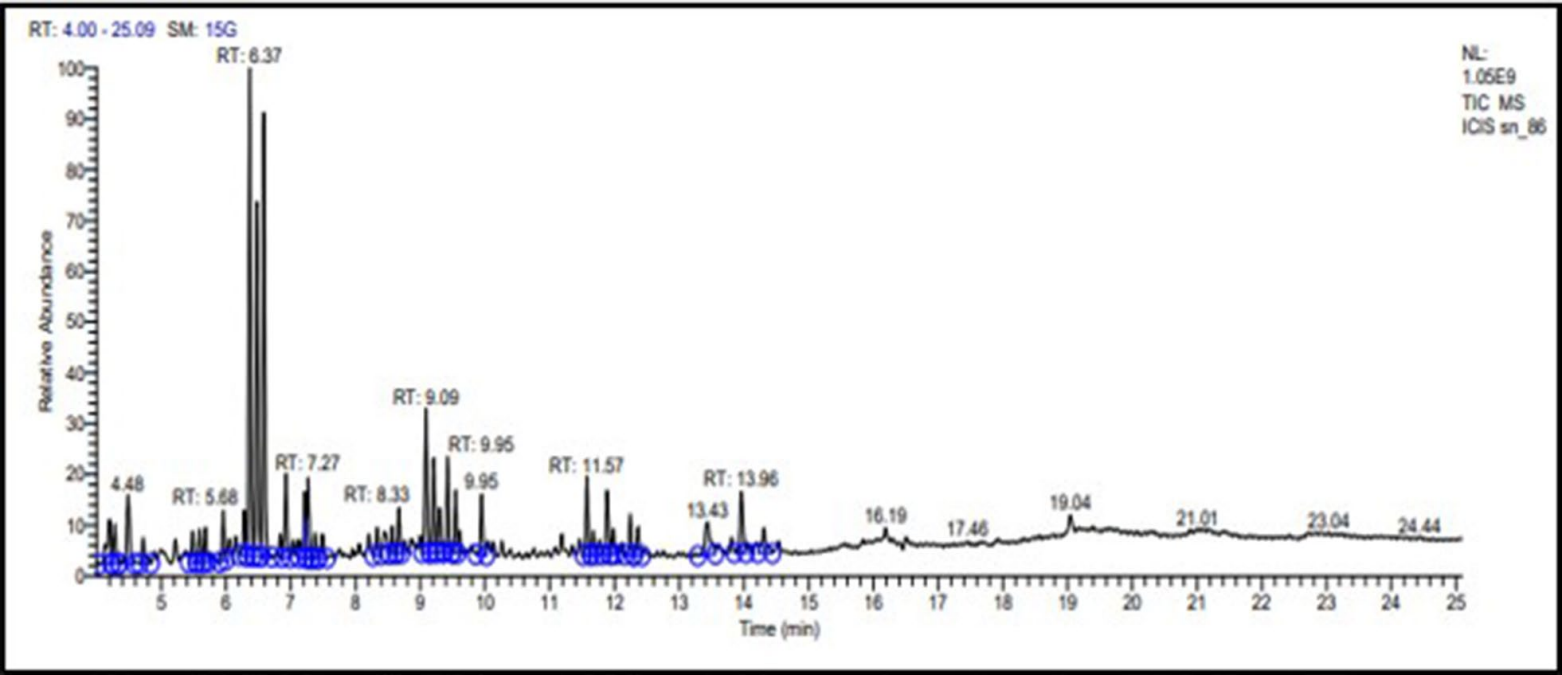
GC–MS Spectrum of EtOAc extract isolated from *Streptomyces* *levis* strain HFM-2

The EtOAc extract obtained from *Streptomyces* *levis* strain HFM-2 was analyzed via GC–MS which revealed the presence of various compounds with number of peaks at different retention times (Fig. 8). Out of thirty detected compounds of GC–MS, five were the major compounds namely 2-Isopropyl-5-methyl-1-heptanol; 3-Octadecene, (E)-; 3- eicosane; 1-Octanol 2-butyl-; and (2,4,6-Trimethylcyclohexyl) methanol (Additional file 1: Fig. S4 and Table 3).

**Table 3:** Major compounds present in EtOAc extract of *Streptomyces* *levis* strain HFM-2 identified by GC–MS analysis

S. No.	Compounds	Retention time	Area %	Molecular weight	Molecular formula	Biological activity	References
1	2-Isopropyl-5-methyl-1-heptanol	6.37	14.94	172.31	C_11_H_24_O	Antimicrobial	Mannaa and Kim (2018); Jha et al. (2022)
2	3-Octadecene, (E)-	6.37	14.94	252.48	C_18_H_36_	Antimicrobial, Antiplasmodial	Balachandar et al. (2018);Tambunan et al. (2020)
3	3-Eicosene, (E)-	6.48	10.98	280.53	C_20_H_40_	Antifungal,Antioxidant, Cytotoxic	Banakar and Jayara j (2018); Marimuthu et al. (2020)
4	1-Octanol, 2-butyl-	6.59	13.41	186.33	C_12_H_26_O	Antimicrobial	Muthulakshmi et al. (2012)
5	(2,4,6-Trimethylcyclohexyl) methanol	4.48	3.31	156.26	C_10_H_20_O	–	Sarker (2011)

## Discussion

Bioactive substances found in nature have always been interesting, particularly those produced by microorganisms because of their complex chemical composition and extremely precise biological functions (Lee et al. 2018). Actinobacteria, the largest phylum of bacteria kingdom, has received major focus from scientific community (Anandan et al. 2016). To increase the pool of actinobacterial diversity available for screening, isolation of new types from different environments has been required. Phylum actinobacteria contains the largest genus *Streptomyces,* that resides primarily in soil (Shah et al. 2016; Kemung et al. 2018), and also present as common colonizers of human bodies, mainly genital tract, respiratory tract and human skin, as detected using molecular techniques (Herbrík et al. 2020; Bolourian and Mojtahedi 2018; Corretto et al. 2021). In several studies, *Streptomyces* spp. also have been reported on fishes, termite gut, and goat feces (Tan et al. 2009; Li et al. 2020; Boxberger et al. 2020). In current study, an active strain of *Streptomyces* (HFM-2), with potent antioxidant, anticancer, and antimicrobial activity, has been identified from human gut. *Streptomyces* strain HFM-2 was characterized through the polyphasic approach that included cultural, physiological, morphological, biochemical, chemotaxonomical, molecular characterization, and phylogenetic analysis. The morphological observations revealed that *Streptomyces* strain HFM-2 produced dark pink substrate mycelium and light pink aerial mycelium bearing spira-type spore chains having smooth spore surface. Morphological and chemotaxonomical properties (LL-DAP in cell wall and no specific sugar in hydrolysate of whole cell) were typical of the genus *Streptomyces* and suggested that strain HFM-2 belongs to the genus *Streptomyces*. The phylogenetic analysis, showed similarity of 98.75%-100% with various *Streptomyces* spp. and the maximum sequence similarity (100%) with *Streptomyces* *levis* NBRC 15423 (T) strain. Phylogenetic tree confirmed its similarity with *Streptomyces* *levis* NBRC 15423 (T) due to distinct clade formation at the bootstrap value of 63% i.e. potentially higher than the threshold value of 50%. The strain could survive and tolerate NaCl concentrations up to 2%, temperature up to 42 °C, and pH up to 11.0. The physiological and metabolic activities of bacteria significantly require enzymes for breakdown of macromolecules facilitating growth and development (Daigham and Mahfouz 2020; Kumar et al. 2020). The strain HFM-2 produces enzymes like amylase, catalse, urease, citrase etc. The results revealed that HFM-2 could utilize various carbon sources like glucose, arabinose, xylose, rhamnose, and raffinose. As far as we are aware, no strain of *Streptomyces* *levis* has been identified from human gut microbiota so far. In addition, in the present study *Streptomyces* *levis* strain HFM-2 is being reported for the antioxidant, anticancer and DNA damage protective potential.

ROS are unstable, reactive, partially reduced oxygen derivatives produced as byproduct of normal metabolic processes.They include hydrogen peroxide (H_2_O_2_), superoxide anion (O_2_^**·**−^), singlet oxygen (^1^O_2_), and hydroxyl radical (^**·**^OH), that act as secondary messengers in cell signaling, also required for various biological processes in both normal and cancer cells (Yang et al. 2018). In response to oxidative stress, the human body has antioxidant mechanisms that play important role in neutralization of free radicals and detoxification of oxidative stress damage (Sharma et al. 2022). A variety of plants and microbes have been reported to exhibit antioxidant activities (Djebbah et al. 2022). Antioxidants protect the body from the harmful free radicals during metabolic processes. As a result, they help to prevent arthritis, cancer, diabetes, atherosclerosis, inflammation and neurodegenerative disorders etc. (Gautham and Onkarappa 2013; Law et al. 2019). There are several in vitro assays used to measure antioxidant potential, and each of these has its own limitations. It is strongly recommended to use multiple assays to determine antioxidant potential because each assay can assess the antioxidant potential for one particular reaction system (Kasote et al. 2015). In the present study five assays exhibiting different mechanism of action were performed to estimate the overall antioxidant potential of EtOAc extract of *Streptomyces* *levis* strain HFM-2. These assays revealed the DPPH, ABTS, superoxide anion radical scavenging activities, and molybdate and ferric ions reducing powers of the EtOAc extract. Based on the formation of the non-radical form (DPPH-H) during the reaction, DPPH assay works by reducing DPPH in an alcoholic solution in the presence of an extract that donates hydrogen. When DPPH radicals come into contact with a proton-donor substrate, such as an antioxidant, the radicals are scavenged and the absorbance decreases. Antioxidants reduce the deep purple chromogen radical (DPPH·) to the corresponding pale yellow hydrazine (Gulcin 2020).

Many researchers have also documented the DPPH free radical scavenging activity of different *Streptomyces* spp., however the reported IC_50_ values and concentrations were higher in comparison to *Streptomyces* *levis* strain HFM-2. In the present study, EtOAc extract displayed high DPPH free radical scavenging potential i.e. 64.76 ± 0.13% at low concentration i.e. 600 µg/mL. The deep purple to yellow colored diphenylpicrylhydrazine change was observed, indicating hydrogen donating ability of EtOAc extract. The 50% (IC_50_ value) free radical scavenging potential of EtOAc extract was obtained at 497.19 µg/mL. However, Tan et al. (2015) exhibited hydrogen donating ability of methanolic extract of *Streptomyces* strain MUM256 with 12.08 ± 1.05% scavenging activity at high concentration i.e. 4000 µg/mL. Siddharth & Vittal (2018) reported moderate scavenging activity (i.e. 56.55 ± 3.1%) of partially purified extract from *Streptomyces* sp. S2A against DPPH free radicals at 1000 µg/mL, and IC_50_ value of 0.86 mg/mL. Another study illustrated 65.87 ± 2.55% DPPH scavenging activity in the ethyl acetate extract of the *Streptomyces* sp. GLD25 at a very high concentration i.e. 30 mg/mL, with higher IC _50_ value i.e. 20.2 ± 2.05 mg/mL (Djebbah et al. 2022).

The reactions with ABTS^+^ radicals involve both Hydrogen Atom Transfer (HAT) and Single Electron Transfer (SET), considered as more reactive than DPPH free radicals, resulting in decolorization of bluish-green colors. The presence of peroxodisulfate accelerates the production of ABTS + , which is typically produced by chemical reaction of K_2_S_2_O_8_ as an oxidant (Badarinath et al. 2010; Gulcin 2020; Salehi et al. 2020). Tan et al. (2017) and Kemung et al. (2020) reported ABTS free radical reduction potential of extracts from *Streptomyces* sp. strain MUM212 and *Streptomyces* sp.strain MUSC14 with 61.52 ± 3.13 and 62.71 ± 3.30% scavenging activity, respectively at very high concentration (4000 µg/mL). However, in the present study, EtOAc extract of *Streptomyces* *levis* HFM-2 exhibited higher ABTS free radical scavenging potential (P ≤ 0.05) with 69.53 ± 0.19% inhibition at 600 µg/mL, and IC_50_ value of 388.13 µg/mL.

The active free radicals also have precursors such as superoxide anion having potential to react with biological macromolecules and causing tissue damage. Superoxide anion (O_2_^**·**−^) is a one-electron-reduced form of molecular oxygen that produces harmfull hydroxyl radicals (^**·**^OH) and singlet oxygen (^1^O_2_), both of which contribute to oxidative stress (Karadag et al. 2009). In current study, EtOAc extract exhibited potent superoxide anion scavenging property with 84.82 ± 0.21% inhibition at 600 µg/mL. The lower IC_50_ value i.e. 268.79 µg/mL indicated significant inhibitory activity (P ≤ 0.05) of the extract from *Streptomyces* *levis* strain HFM-2. Superoxide anion radical causes reduction of NBT producing blue formazan, EtOAc extract inhibited the reduction of NBT which caused decrease in absorbance. Same trend was also reported by Parejo et al. (2002). Similarly, Kumar et al. (2014) demonstrated the superoxide anions scavenging activity of ethyl acetate extract from *Streptomyces* *lavendulae* strain SCA5 at high IC_50_ valuei.e 864.71 ± 1.15 μg/mL. In our previous study, Rani et al. (2018) showed 82.08 ± 0.93% scavenging activity at 5 mg/mL of EtOAc extract from *Streptomyces* *cellulosae* strain TES17 against superoxide anion scavenging, and IC_50_ value at 2.41 mg/mL.

The EtOAc extract of *Streptomyces* *levis* strain HFM-2 demonstrated promising antioxidant activities with phosphomolybdenum and ferric-reducing power assays that employ single electron transfer (Koksal and Gulcin 2008). FRAP assay is based on reduction of colorless Fe^3^ + -2,4,6-tripyridyl-s-triazine complex by the action of electron-donating antioxidants to the intense blue Fe^2^ + -2,4,6-tripyridyl-s-triazine complex in acidic medium. The high reduction potential of extracts/compounds shows highy absorbance and intensity (Floegel et al. 2011). In current study, total reducing power of EtOAc extract was assessed by intense blue color. The ferric reducing power of EtOAc extract showed significant absorbance and 856.83 ± 0.76 µg as Ascorbic Acid Equivalents (AAE)/mg dry weight of theextract.

The total antioxidant assay was developed for the quantitative determination of antioxidant capacity, through the formation of phosphomolybdenum complex. The assay is based on the reduction of Mo^6^ + to Mo^5^ + by the sample analyte producing green color (Alam et al. 2013). The molybdate reducing ability of EtOAc extract was directly proportional to absorbance. The phosphomolybdate ion reducing power of EtOAc extract showed significant absorbance and 860.06 ± 0.01 µg/mL as Ascorbic Acid Equivalents (AAE)/mg dry weight of the extract. The reducing capacity of EtOAc extract is good indicator of its potential antioxidant activity. Gautham and Onkarappa (2013) reported 0.51 absorbance units at 700 nm (88.69 µg as ascorbic acid equivalent) at 200 µg of n-butanol extract of *Streptomyces* *fradiae* strain GOS_1_. Similary, Fahmy and Abdel-Tawab (2021) demonstrated 7.70 ± 0.03 and 7.67 ± 0.15 µg phosphomolybdate and ferric ion reducing power, respectively as ascorbic acid equivalent in 150 µg EtOAc of *Streptomyces* sp. NMF6.

The majority of natural antioxidants are phenolic compounds, with flavonoids, phenolic acids, tocopherols being the most important groups (Gulcin 2020). Phenolic acids are derivatives of hydroxycinnamic acids and hydroxybenzoic acids. Total phenolic content (TPC) was determined as gallic acid or another putative phenolic compound equivalent. The high quantity of phenolic content was associated with high antioxidant ability. Hence, this assay is important parameter for the determination of total antioxidant capacity (Terpinc et al. 2011; Gulcin 2020). The total phenolic and flavonoid content in the EtOAc extract suggested that phenolic compounds made a significant contribution to the antioxidant potential of EtOAc extract of *Streptomyces* *levis* strain HFM-2. Second class of phenolic compounds are flavonoids characterized with phenolic rings. Both phenolics and other flavonoids act as antioxidants and scavenging free radicals (Rani et al. 2018; Gulcin 2020). The total phenolic content, expressed as GAE, of EtOAc extract was measured as 77.63 ± 0.02 µg GAE/mg of dry extract. Flavonoids, along with phenolic acids are the most important secondary metabolites, which were quantified by colorimetric assay. The total flavonoid content, expressed as catechin, of EtOAc extract was observed as 71.25 ± 0.02 µg catechin/mg of dry extract demonstrating significant indicator of its potential antioxidant activity.However, further confirmation of phenolic compounds in EtOAc extract was done by UHPLC analysis based on the retention time with compounds such as catechin, epicatechin, caffeic acid, chlorogenic acid, gallic acid and tert-Butyl hydroquinone. Tert-butyl-hydroquinone (TBHQ) and Gallic acid are common types of antioxidant compounds having potent antibacterial, antifungal, and antiviral properties (Wieczorek et al. 2016; Anoop et al. 2022). According to UPLC analysis, the tert-butyl hydroquinone, and gallic acid were the major phenolic compounds in the EtOAc extract responsible for various bioactivities.

In order to support the antioxidant potential, DNA protecting effect of EtOAc extract was evaluated, utilizing DNA damage model induced by oxidative stress. Hydroxyl radicals produced from Fenton’s reagent cleave the native supercoiled plasmid DNA into single stranded nicked or linear forms (Kaur and Arora 2017). In the present investigation, simultaneous decrease of single stranded nicked (Form II) and linear forms of DNA (Form III) along with increasing native supercoiled form (Form I) i.e. 21.14%, 29.46%, 30.49% 31.49%, and 37.75% in the presence of EtOAc extract at 2–10 μg/mL, respectively were observed, confirming its protective effect at low concentration. In contrast, in another report, EA extract of *Streptomyces* sp VITSTK7 partially protected the DNA damaged by UV-induced photolysis of H_2_O_2_ at high concentration i.e. 50 μg/mL (Thenmozhi and Kannabiran 2012).

Cancer progression can be initiated by oxidative stress via biological molecule modifications that enhance rate of mutation (Taechowisan et al. 2021). EtOAc extract’s growth inhibitory activity was observed in HeLa cancer cells. In this study, EtOAc extract displayed significant cytotoxicity against HeLa cancer cells with 85.83 ± 0.50% inhibition at concentration of 500 µg/mL, and the IC_50_ value was observed as 50.69 µg/mL. Similarly, Davies et al. (2019) reported cytotoxic inhibition of HeLa cancer cells by extract from *Streptomyces* *albus* UL7B. However, the IC_50_ value was achieved at very high level i.e. 2.277 mg/mL. In a recent report, ethyl acetate extract of strain *Streptomyces* *cangkringensis* exhibited inhibition (71.01 ± 0.24%) and IC_50_ value against HeLa cancer cells at high concentrations i.e. 1000 µg/mL and 410.5 µg/mL, respectively (Saraswathi et al. 2020).

The largest organ of human body is skin that serves as first line of defence for environmental threats (Meglio et al. 2011). Skin barriers are most sensitive due to their constant exposure to sun damage, harmful agents etc. resulting in skin carcinogenesis that enhances cancer initiation, promotion and progression (Ng et al. 2018). Mohansrinivasan et al. (2015) analyzed the petroleum ether extract from grape seeds for cytotoxicity against skin cancer cell line A431 and the IC_50_ value was found at 480 µg/mL. In contrast, the EtOAc extract of *Streptomyces* *levis* HFM-2 caused inhibition of 78 ± 0.5% at 500 µg/mL and very low IC_50_ value i.e. 84.07 µg/mL.

In cancer research EAC carcinoma cells are used worldwide as experimental tumor models (Ozaslan et al. 2011). In this study, EAC cells inhibited by EtOAc extract with 68.59 ± 0.03% inhibition and the IC_50_ value was found at low conentration i.e. 164.91 µg/mL. The increasing concentration of EtOAc extract increases cytotoxicity against tumor cells with a significance difference (p ≤ 0.05). An ideal chemoprotective drug requires high specificity that enables it to distinguish between normal cells and cancer cells (Ser et al. 2015a, b). Immense efforts have been devoted to search for novel chemotherapeutic drugs having high specificity and potential. In current study, investigation of specificity of *Streptomyces* *levis* strain HFM-2 revealed that EtOAc extract was nontoxic against L929 normal cells when compared to HeLa, A431 skin, and EAC carcinoma cells. These significant results have revealed new insight into EtOAc extract cytotoxic potential against different cancer cells, primarily HeLa cancer cells with high specificity. So, the *Streptomyces* *levis* strain HFM-2 could be helpful as producer strain of anticancer drugs in future.

Brightfield and fluorescence microscopies were used to observed cellular morphological changes of HeLa cancer cells (Zhang et al. 2017). The significant results of this study indicate various extents of cytotoxic effects on HeLa cells in the presence of EtOAc extract. Based on the results, EtOAc extract of HFM-2 could be utilized as apoptosis inducing anticancer agent. AO/EtBr staining of HeLa cancer cells treated with EtOAc extract showed morphological changes such as condensed nuclei, membrane blebbing and apoptotic bodies. AO/Etbr fluorescence based live/dead assay was used to evaluate primarily the proportion of live and dead cells in HeLa cancer cells treated with EtOAc extract (Prashanthi et al. 2015). The findings of DAPI staining exhibited several changes in nuclear morphology like chromatin condensation, shrinkage, loss of nuclear architecture and inter nucleosomal DNA fragmentation which ultimately lead to apoptotic cell death (Manivasagan and Oh 2015). These changes were observed in HeLa cancer cells when treated with EtOAc extract of HFM-2.

Cancer cell behaviour is affected by oxidative stress-mediated signalling events. The rapid metabolic activities and increased signalling pathways of cancer cells generate significantly more ROS than normal cells (Alapati and Handanahal 2021). If ROS concentrations exceed the levels that are incompatible with cellular survival, it causes cytotoxic effect, cell cycle arrest, apoptosis, necrosis, and limiting cancer progression (Tian et al. 2020). The EtOAc extract produced by *Streptomyces* *levis* strain HFM-2 inhibited mitochondrial electron transport chain that increased ROS in HeLa cancer cells as detected by DCFH-DA (fluorescent) dye (Woo et al. 2007). Mitochondrial ATP synthesis is based on mitochondrial membrane potential (ΔΨM), an essential component of proton motive force (Δ p). This potential acts as a relative measure which detect cell stress/apoptosis status by florescence dye Rhodamine 123 and acts as indicator of cell death (Alapati and Handanahal 2021). In this study, the increasing concentration of EtOAc extract resulted in decreasing mitochondrial membrane potential. The inhibition of mitochondrial membrane potential seems to be the common mechanism for cytotoxicity of EtOAc extract. Similarly, Bajić et al. (2013) observed that mitochondrial membrane potential loss was triggered by extract and initiating apoptosis in concentration and time-dependent manner.

The results of both the antioxidant assays and in vitro anticancer screening suggested potential biologically active compounds' presence in EtOAc extract which further lead towards chemical analysis employing GC–MS for identification of chemical constituents of EtOAc extract. GC–MS analysis revealed the presence of various compounds including, 2-Isopropyl-5-methyl-1-heptanol; 1-Octanol, 2-butyl-, 3-Octadecene, (E), and 3-Eicosene, (E) in the EtOAc extract of *Streptomyces* *levis* strain HFM-2. These compounds are known to possess antimicrobial, antioxidant and anticancer activities as per previous reports (Muthulakshmi et al. 2012; Banakar and Jayaraj 2018; Lulamba et al. 2021; Elsayed et al. 2020). Among these compounds, 1-octanol, 2-butyl and 2-isopropyl-5-methyl-1-heptanol, exhibiting antimicrobial activity, were detected in the crude extract of *Feronia* *elephantum,* crude metabolic extract from *Streptomyces* *lavendulae* M3, and *Pseudomonas* protegens AS15 (Muthulakshmi, et al. 2012; Mannaa and Kim 2018; Jha et al. 2022). The 3, octadecene (E) has been revealed in the solvent extract of vermicast isolated actinomycete species (Balachandar et al. 2018) whereas, 3-Eicosene (E) was detected in crude extracts of *Photorhabdu* *sheterorhabditis*, *Streptomyces* sp. SLR03, *Chaetomium* *globosum*, an endophytic fungus, and *Streptomyces* sp. strain KF15 displaying antimicrobial activity (Tambunan et al. 2020; Marimuthu et al. 2020; Kaur et al. 2020; Lulamba et al. 2021; Bhat et al. 2022). 

In conclusion, in current study, *Streptomyces* *levis* strain HFM-2 has been identified from healthy human gut. This is a novel source that produces potent secondary metabolites and exhibits several bioactivties including antioxidant, anticancer, and antimicrobial. The EtOAc extract of *Streptomyces* *levis* strain HFM-2 showed the strong free radical scavenging potential like ABTS, DPPH, Superoxide anion scavangeing activities, and also protected from oxidative stress induced DNA damage. EtOAc extract exhibited *in-vitro* cytotoxic activity for various cancer cell lines and nontoxicity to normal cell line. In addition, EtOAc extract induced depolarization of mitochondrial membrane potential by increasing ROS generation and DNA fragmentation of HeLa cervical cancer cells, resulting in apoptosis. In EtOAc extract four major chemical compounds were identified by GC–MS analysis. Further analysis by UPLC demonstrated phenolic compounds which might be responsible for various bioactivities Thus, the study concludes with observation that EtOAc extract could be utilized for bioactive drugs development in medical and pharmaceutical applications.

## Supplementary Information

**Additional** **file** **1:** **Figure** **S1.** Cultural characteristics of *Streptomyces* *levis* strain HFM-2 on ISPmedia showingaerial mycelium andsubstrate mycelium. **Figure** **S2.** Phylogenetic tree obtained by the neighbour-joining algorithm based on complete 16S rRNA gene sequences of *Streptomyces* spp. showing the position of *Streptomyces* *levis* strain HFM-2. Bootstrap valuesare shown at the nodes. **Figure** **S3.** Densitometric analysis of DNA protective effects of *Streptomyces* *levis* strain HFM-2 extract in the presence of hydroxyl radicals generated in DNA nicking assay. Form I-supercoiled DNA, Form II- single-stranded nicked DNA, Form III- linear DNA. **Figure** **S4.** Chemical structures of different bioactive compounds detected in GC-MS analysis. **Table** **S1.** Cultural features of *Streptomyces* strain HFM-2 on different media. **Table** **S2.** Antioxidant activity of EtOAc extract from *Streptomyces* *levis* strain HFM-2.

## Data Availability

All data generated or analyzed during this study are included in this article.

## Abbreviations

SCNA: Starch casein nitrat agar
ISP: International *Streptomyces* Project
DPPH: (2,2-Diphenyl-1-picrylhydrazyl)
ABTS: 2,2′-Azino-bis (3-ethylbenzothiazoline-6-sulfonic acid)
FRAP: Feric reducing antioxidant potential
PMS: Phenazine methosulphate
NADH: Nicotinamide adenine dinucleotide
NBT: Nitro blue tetrazolium
GC-MS: Gas-Chromatography-Mas-Spectroscopy
DMEM: Dulbecco’s modified Eagle’s medium
