# Supplementary material for: Antioxidant and chemoprotective potential of Streptomyces levis strain isolated from human gut
Source: AMB Express. 2023 Jul 7;13:69. doi: 10.1186/s13568-023-01570-7 (PMC10328884; doi:10.1186/s13568-023-01570-7)
Supplement: Supplementary file 1 — Additional file 1: Figure S1. Cultural characteristics of Streptomyces levis strain HFM-2 on ISPmedia showingaerial mycelium andsubstrate mycelium. Figure S2. Phylogenetic tree obtained by the neighbour-joining algorithm based on complete 16S rRNA gene sequences of Streptomyces spp. showing the position of Streptomyces levis strain HFM-2. Bootstrap valuesare shown at the nodes. Figure S3. Densitometric analysis of DNA protective effects of Streptomyces levis strain HFM-2 extract in the presence of hydroxyl radicals generated in DNA nicking assay. Form I-supercoiled DNA, Form II- single-stranded nicked DNA, Form III- linear DNA. Figure S4. Chemical structures of different bioactive compounds detected in GC-MS analysis. Table S1. Cultural features of Streptomyces strain HFM-2 on different media. Table S2. Antioxidant activity of EtOAc extract from Streptomyces levis strain HFM-2. [file 13568_2023_1570_MOESM1_ESM.docx]

**Antioxidant and Chemoprotective Potential of *Streptomyces levis* Strain Isolated from Human Gut**

**Jaya Verma^1^, Shivani Attri^2^, Saroj Arora^2^, Rajesh Kumari Manhas^1*^**

**^1^Department of Microbiology, Guru Nanak Dev University, Amritsar, Punjab, India**

**^2^Department of Botanical & Environmental Sciences, Guru Nanak Dev University, Amritsar, Punjab, India**

****Corresponding author*: Email:** **rkmanhas@rediffmail.com**

**
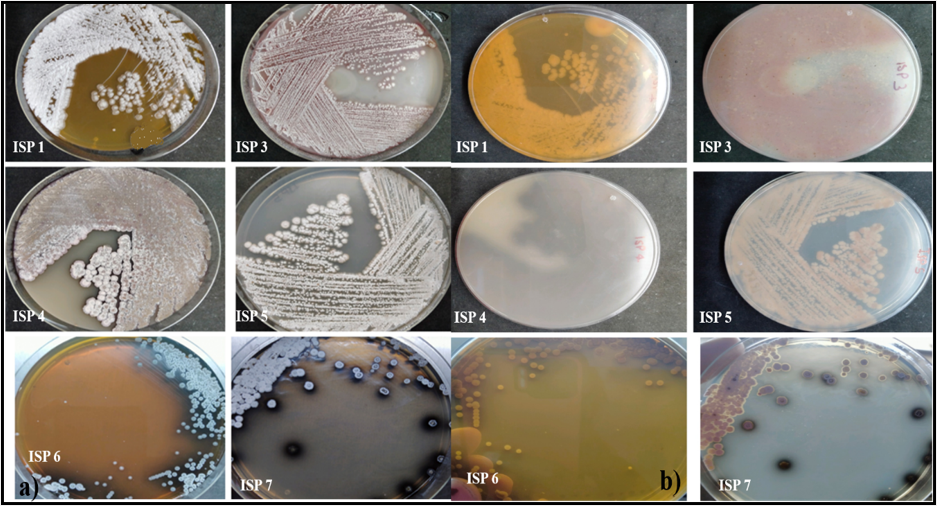
**

**Figure S1: Cultural characteristics of *Streptomyces levis* strain HFM-2 on ISP (International *Streptomyces* Project) media showing (a) aerial mycelium and (b) substrate mycelium.**


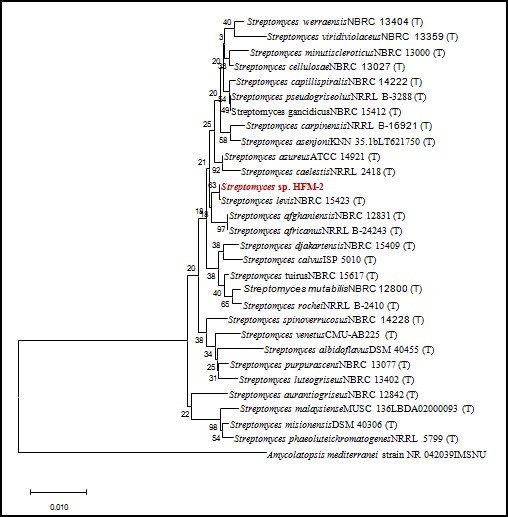


**Figure S2: Phylogenetic tree obtained by the neighbour-joining algorithm based on complete 16S rRNA gene sequences of *Streptomyces* spp. showing the position of *Streptomyces levis* strain HFM-2. Bootstrap values (expressed as percentage of 1000 replications) are shown at the nodes.**


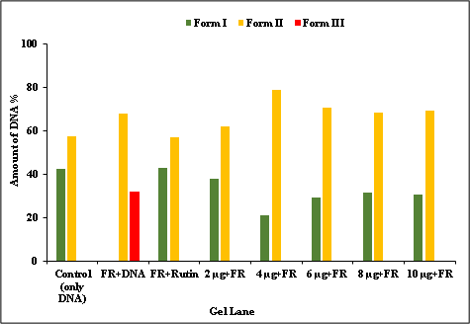


**Figure S3: Densitometric analysis of DNA protective effects of *Streptomyces levis* strain HFM-2 extract in the presence of hydroxyl radicals generated in DNA nicking assay. Form I-supercoiled DNA, Form II- single-stranded nicked DNA, Form III- linear DNA.**

**
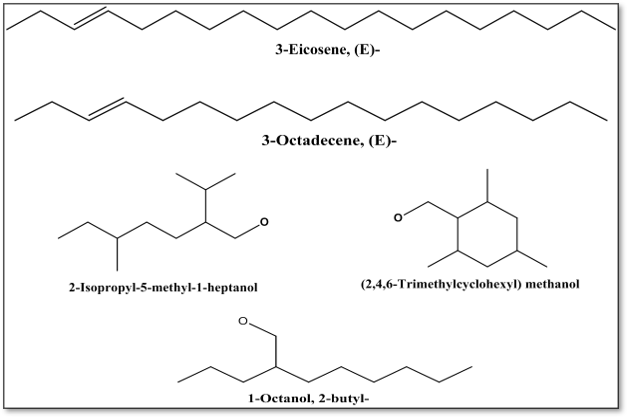
**

**Figure S4: Chemical structures of different bioactive compounds detected in GC-MS analysis.**

| **Media** | **Sporulation** | **Substrate mycelium** | **Aerial mycelium** | **Growth** | **Pigmentation** |
| --- | --- | --- | --- | --- | --- |
| **ISP1** | Silver white | Pale yellow | Silver white | Good | - |
| **ISP2** | Pale yellow | Pale yellow | Grey | Very Poor | - |
| **ISP3** | White | Pink | White | Good |  |
| **ISP4** | Reddish cinnamon | Light brown | Reddish cinnamon | Good | - |
| **ISP5** | White | Cream | White | Good | - |
| **ISP6** | Grey | Dark black | Grey | Good | Melanin |
| **ISP7** | Off white | Dark grey | Off white | Good | Melanin |
| **SCNA** | White | Dark pink | Light pink | Good | **-** |

**Table S1:Cultural features of *Streptomyces* strain HFM-2 on different media:**

**Present = (+), absent (-)**

**Table S2: Antioxidant activity of EtOAc extract from *Streptomyces levis* strain HFM-2**

| **Concentration of EtOAc (µg/mL)** | **Antioxidant activity** | | |
| --- | --- | --- | --- |
|  | **DPPH radical scavenging activity (%)** | **ABTS radical scavenging activity (%)** | **Superoxide anion scavenging assay (%)** |
| 100 | 21.11 ± 0.11^f^ | 14.50 ± 0.15 ^f^ | 18.10 ± 0.23 ^f^ |
| 200 | 35.23 ± 0.13 ^e^ | 28.55 ± 0.96 ^e^ | 30.72 ± 0.21^e^ |
| 300 | 43.20 ± 0.13 ^d^ | 38.79 ± 0.19 ^d^ | 50.95 ± 0.21 ^d^ |
| 400 | 46.91 ± 0.12 ^c^ | 51.84 ± 0.20 ^c^ | 69.00 ± 0.25 ^c^ |
| 500 | 51.64 ± 0.11^b^ | 56.84 ± 0.17 ^b^ | 80.19 ± 0.31 ^b^ |
| 600 | 64.76 ± 0.13^a^ | 69.53 ± 0.19 ^a^ | 84.82 ± 0.21 ^a^ |

**Values represented as ±SD, at the level of p < 0.05 significant difference among them.**
